# Supplementary material for: Effects of Two Trichoderma Strains on Apple Replant Disease Suppression and Plant Growth Stimulation
Source: J Fungi (Basel). 2024 Nov 20;10(11):804. doi: 10.3390/jof10110804 (PMC11595690; doi:10.3390/jof10110804)
Supplement: Supplementary file 1 [file jof-10-00804-s001.zip › jof-3246465-supplementary.pdf]

Table S1 Inhibition effect of *Trichoderma* on the growth of mung bean.

***Trichoderma* spore suspension concentration (spores/mL)**	**M19**			**L7**		
	**Radicle length(cm)**	**Fiber root number**	**Incidence rate**	**Radicle length(cm)**	**Fiber root number**	**Incidence rate**
1×10^5	7.86±0.62a	8.47±0.79ab	33.33	10.59±0.29b	8.93±0.41b	40.00
1×10^6	10.57±0.83b	10.33±0.84b	13.33	12.11±0.52b	11.13±0.41c	13.33
1×10^7	7.29±1.85a	6.73±1.07a	33.33	6.23±0.63a	9.27±0.07b	20.00
HS2	5.43±0.62a	6.47±0.68a	100.00	5.43±0.62a	6.47±0.68a	100
CK	6.90±0.48a	8.47±0.29ab	0.00	6.90±0.48a	8.47±0.29b	0.00

Table S2 Collecting soil information from two *Trichoderma* strains

Number	*Trichoderma* strain	Origin	Soil type	Location
1	L7	Rhizosphere soil	Red Soil	Yunnan Province
2	M19	Rhizosphere soil	Red Soil	Yunnan Province

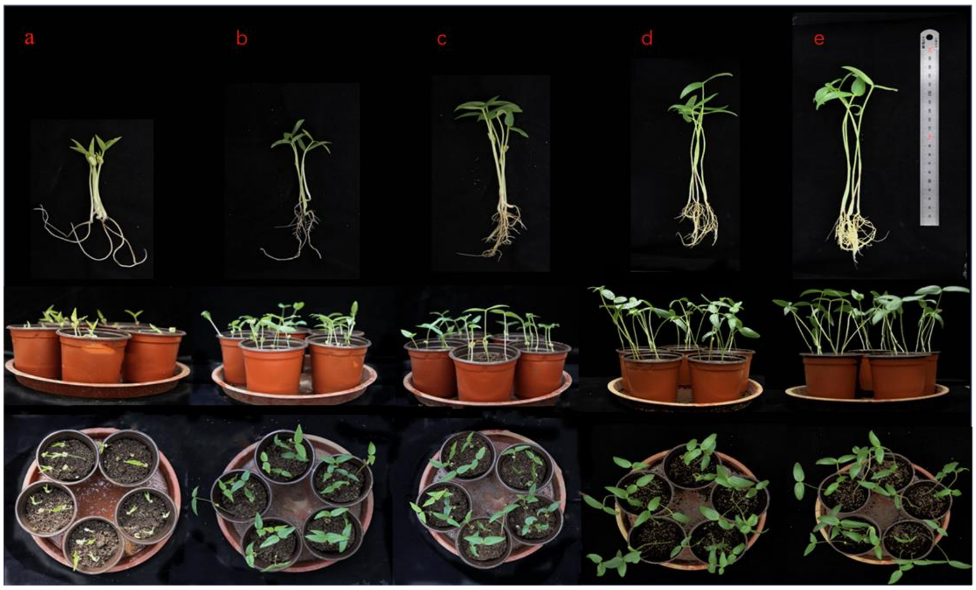

Figure S1 Effect of *Trichoderma* on the growth of mung bean. a-b-c-d-e refers to CK, L7 soaking, M19 soaking, L7 soaking+irrigation root, M19 soaking+irrigation root, respectively

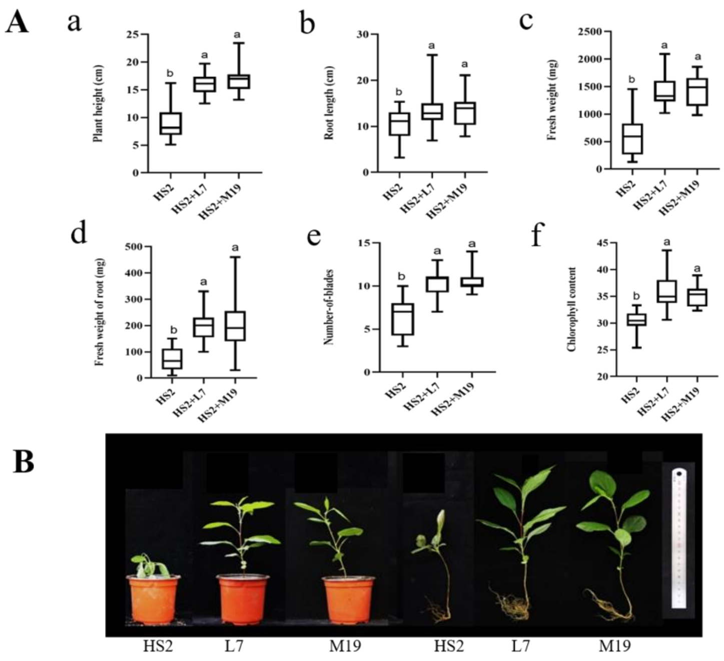

Figure S2 Effect of *Trichoderma* and *F. oxysporum* co-treatment on various physiological indicators of *M. robusta* plants. (A) Determination of physiological indexes of *Trichoderma* on disease prevention of *M. robusta*. a-b-c-d-e-f represents plant height, root length, number of leaves, fresh weight, chlorophyll content, root fresh weight. *M. robusta* seedling parameters including plant height, fresh weight, root length, fresh root weight, leaf number and chlorophyll content improved after treatment with *Trichoderma* spore suspensions in diseased soil. Values with superscript letters a and b are significanty diferent across columns (P<0.05). (B) The effect of *Trichoderma* on the growth of *M. robusta*. HS2, HS2+L7 and HS2+M19 respectively represent: *F. oxysporum* HS2 treated *M. robusta* seedlings; *F. oxysporum* HS2+M19 co treated M. robusta seedlings; *F. oxysporum* HS2+L7 co treated *M. robusta* seedlings.

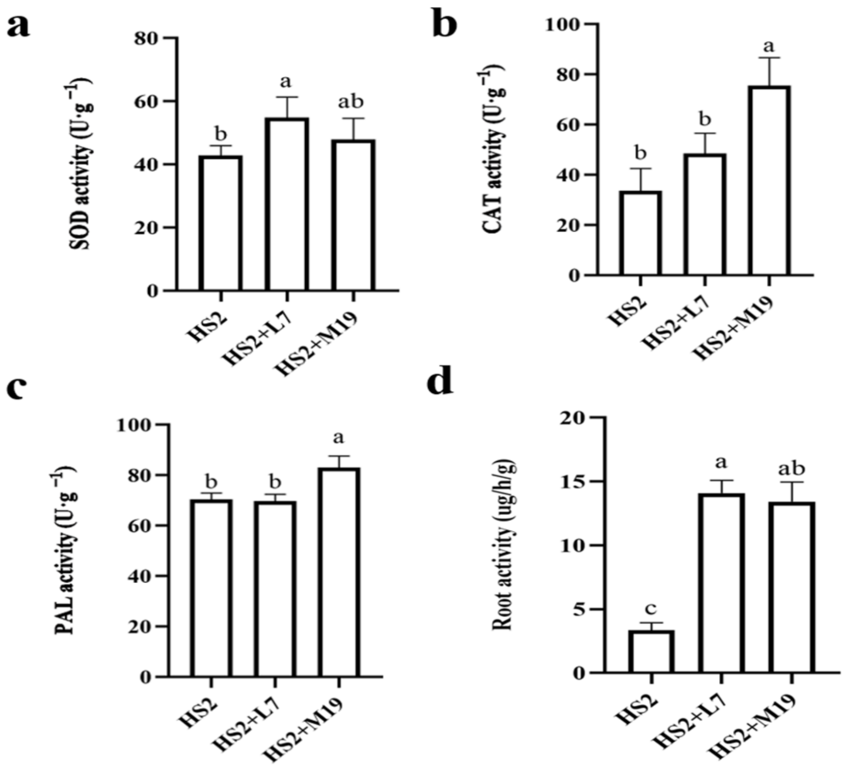

Figure S3 The Effect of *Trichoderma* and *F. oxysporum* co treatment on activity of defense enzymes in the roots of *M. robusta* seedlings. a-b-c-d represents SOD activity, CAT activity, PAL activity, and root activity. Values with superscript letters a, b and c are significanty diferent across columns (P<0.05).

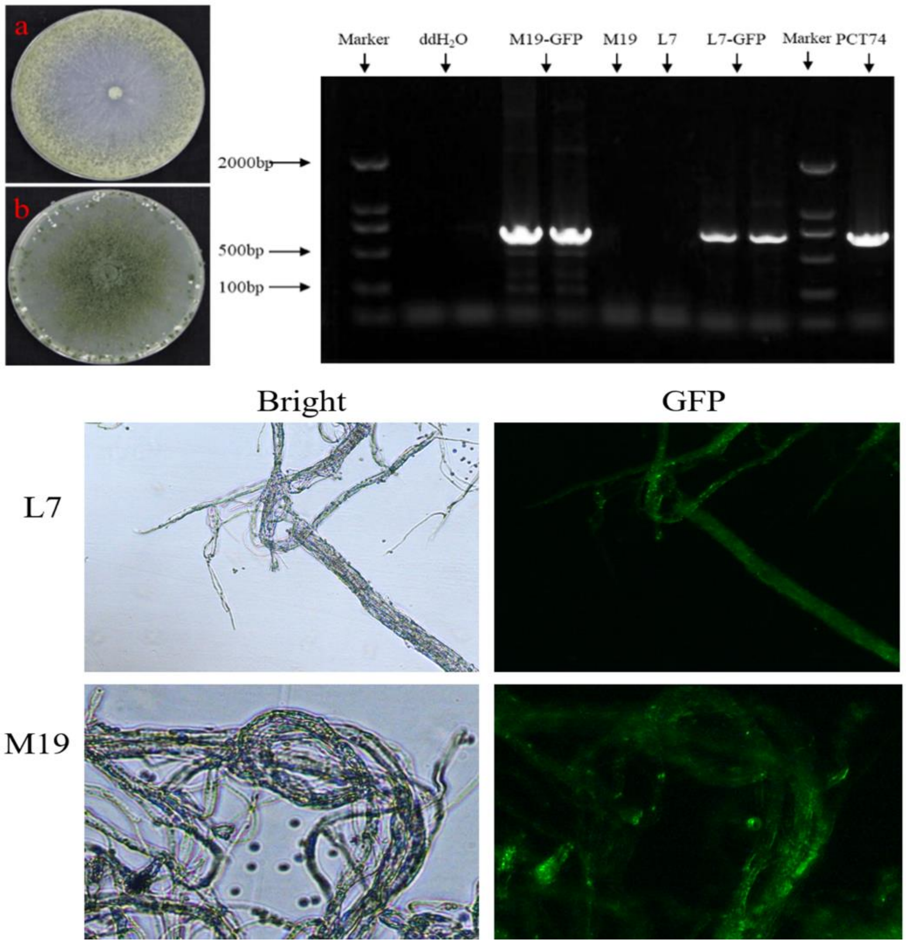

Figure S4 Colony morphology, PCR detection and fluorescence observation of mycelium results of two transformants. a and b represent L7 colony, M19 colony.
